# Cross-Sectional Study of Chronic Obstructive Pulmonary Disease Prevalence Among Smokers, Ex-Smokers, and Never-Smokers in Almaty, Kazakhstan: Study Protocol

**DOI:** 10.2196/resprot.7422

**Published:** 2017-07-25

**Authors:** Almaz Sharman, Baurzhan Zhussupov, Dana Sharman, Assel Stambekova, Sattar Yeraliyev

**Affiliations:** ^1^ Kazakhstan Academy оf Preventive Medicine Almaty Kazakhstan

**Keywords:** COPD, Kazakhstan, cross-sectional, study protocol, tobacco smoking, risk factors

## Abstract

**Background:**

Chronic obstructive pulmonary disease (COPD) is significantly underdiagnosed in Kazakhstan, and there is no previously conducted study on COPD prevalence in the country.

**Objective:**

The purpose of this study is to assess the prevalence of COPD among individuals aged 40 to 59 years based on results of spirometry before and after bronchodilator, presence of structural changes in the lungs (emphysema, inflammatory changes, and thickening of the walls of the large and small airways) detected by computer tomography, and the symptoms of COPD. The study has 3 study groups: smokers of conventional cigarettes, those who had quit smoking 1 to 5 years ago, and those who haven’t smoked cigarettes.

**Methods:**

This is an observational study with a cross-sectional design among individuals aged 40 to 59 years in Almaty, Kazakhstan. The sample of 900 individuals of both sexes contains 500 smokers, 200 ex-smokers, and 200 never-smokers. Study measures include spirometry, chest computed tomography, electrocardiography, physical exams, laboratory testing of serum, anthropometry, and 6-minute walk test. Data are collected by computer-assisted personal interviewing with tablets. The questionnaire was designed to explore possible COPD risk factors including history of smoking, current smoking, level of smoking exposure (in pack-years), passive smoking, occupational and environmental hazards, and covariates: age, gender, ethnicity, education, occupation, and self-reported morbidity. COPD Assessment Test (CAT) is used to collect information about COPD symptoms.

**Results:**

We have completed the participant recruitment and study procedures. Currently, we are working on data processing and data analysis. The authors anticipate the preliminary results should be available by September 2017. Study results will be published in peer-reviewed scientific journals.

**Conclusions:**

This is the first study in Kazakhstan that assesses prevalence of COPD and its comorbidities in the adult population aged 40 to 59 years. The results of the study will be useful for improving COPD preventive measures, better COPD screening, identification, and registration. Findings of the study will also contribute to global knowledge on the epidemiology of COPD.

**Trial Registration:**

ClinicalTrials.gov NCT02926534; https://clinicaltrials.gov/ct2/show/NCT02926534 (Archived by WebCite at http://www.webcitation.org/6rjwGsPOZ)

## Introduction

Chronic obstructive pulmonary disease (COPD) is the fourth leading cause of death worldwide [1]. The number of affected individuals and deaths from COPD are expected to increase as the population ages [2]. In Russia, the largest neighboring country of Kazakhstan, the prevalence of symptomatic COPD in the adult population was estimated to be 15.3% [3]. An estimated 1.4 million individuals in Kazakhstan may be affected by COPD. This estimate is based on studies that have been conducted in other countries in the World Health Organization European Region [4] but not in Kazakhstan, because a study on the prevalence of COPD has not been conducted in the country yet. COPD is significantly underdiagnosed in Kazakhstan. In 2013, the rate of reported COPD cases was 315.9 per 100,000 or around 53,000 registered cases of COPD [5]. A better understanding of the epidemiology and social and other determinants of the disease is needed in order to recognize the true magnitude of the problem and develop effective treatments and prevention strategies.

Various criteria for COPD have been used in population-based studies. The most common criterion is airflow obstruction detected by spirometry testing, defined as a postbronchodilator ratio of forced expiratory volume in 1 second (FEV_1_) to forced vital capacity (FVC) less than 0.7 or 70% [6]. This criterion is simple to implement and widely used in epidemiological surveys. However, COPD prevalence based on this could be slightly biased—overestimated in old subjects and underestimated in younger ones [7]. For this reason, the fifth percentile of the FEV_1_/FVC ratio or the lower limit of normal distribution of the FEV_1_/FVC ratio defined for specific age-gender group is also recommended for use in epidemiological studies [8].

Respiratory symptoms [9] and physical examination [10] were frequently used to evaluate COPD prevalence. However, collecting such data is more important for establishing the clinical diagnosis of COPD [6]. The specific symptoms of COPD include progressive dyspnea, cough, and sputum production [6]. The COPD Assessment Test (CAT) was specially designed to measure how COPD symptoms lead to health status impairment. The test is derived from 8 items, and the score varies from 0 to 40. The cut-off point for the CAT is 10, after which regular treatment for symptoms is recommended [6]. Although a physical examination is rarely used in COPD diagnosis, particularly in detecting mild to moderate COPD, thoracic examination of patients with severe disease can usually reveal the following signs: hyperinflation, wheezing, diffusely decreased breath sounds, hyperresonance on percussion, and prolonged expiration [11].

Chest computed tomography (CT) scan is recommended for subjects with airflow limitation and signs and symptoms suggestive of COPD for making an accurate diagnosis of COPD to exclude other conditions [12]. CT scan also helps to separate COPD patients into 2 main phenotypes, emphysema and small airway disease [13]. CT images can be visually assessed by qualified observers to describe patterns of altered lung structure or quantified for assessment of the extent of emphysema, gas trapping, and airway abnormality [14]. Some studies suggested that severity of emphysema detected by CT scan is associated with greater lung function decline even if airway obstruction is not currently presented [15,16]. As a result, CT-detected emphysema may predict future development of airflow obstruction [16,17].

Tobacco smoking, occupational and environmental exposures including workplace dusts and chemicals, and smoke from home cooking and heating fuels are the main risk factors for COPD [8]. In addition, advanced age, chronic respiratory infections such as tuberculosis, low socioeconomic status, and being underweight may influence COPD development [18,19]. Genetic predisposition also plays an important role in COPD development. Originally described more than 50 years ago, α_1_-antitrypsin deficiency may cause COPD and accounts for 1% to 2% of all COPD cases. There are other genome variants currently being investigated as candidates for COPD genes, but only the Z variant of α_1_-antitrypsin is accepted as a COPD gene at the present time [20].

Comorbidities are frequently found in persons with COPD. Some of them have risk factors, which are the same as for COPD, particularly tobacco smoking and aging. Moreover, systemic inflammation and chronic hypoxia present in COPD patients may cause other health-related conditions. Common comorbidities include heart disease, lung cancer, osteoporosis, metabolic syndrome and diabetes, anemia, anxiety, cognitive decline, and sleep disorders [21,22]. Specific comorbidities increase mortality and poor outcome in COPD, so management of main comorbidities has been included to COPD guidelines [6].

The aim of the study is to assess the prevalence of COPD among smokers, ex-smokers, and never-smokers aged 40 to 59 years based on pulmonary function assessment (spirometry), structural changes (emphysema and large and small airway inflammation with thickening) identified by high-resolution CT, COPD symptoms, and exercise limitations. In addition, the study objectives include comparing the prevalence of health conditions considered as COPD comorbidities (heart disease, hypertension, metabolic syndrome, diabetes mellitus) in 3 study groups and their interaction with COPD.

## Methods

### Study Design

This is an observational study with a cross-sectional design to assess the prevalence of COPD in Almaty, Kazakhstan, among individuals aged 40 to 59 years based on results of spirometry, the presence of structural changes in the lungs, and symptoms of COPD.

The study population is 3 groups of male and female residents of Almaty, the largest city in Kazakhstan, with a population of 1.7 million people, 9% of whom are aged 40 to 59 years. Members of the first group include current smokers with more than a 10 pack-year history of smoking (smokers). The second group comprises individuals who quit smoking from 1 to 5 years ago and have more than a 10 pack-year history of smoking (ex-smokers). The third group comprises persons who have never smoked regularly (ie, smoked less than 100 cigarettes in their lifetime) (never-smokers).

We recruited individuals who are 40 years of age or older because most people are at least 40 years old when the symptoms of COPD first appear [23]. The same age limit is selected for many COPD prevalence studies [24-26]. The upper age was set at 59 years to avoid survival bias [27] possibly leading to underestimating the effects of risk factors on COPD. We have taken into account that the life expectancy at birth among males was only 66 years in Kazakhstan in 2015 [28].

### Inclusion and Exclusion Criteria

Male and female participants aged 40 to 59 years who have a 10 pack-year and more of smoking history (for smokers and ex-smokers) or fewer than 100 cigarettes in their lifetime (for never-smokers) and are able to provide informed consent can be included in this study.

Exclusion criteria:

- Pregnancy
- Fever (37°C or higher) at the time of the visit or during the 2 weeks preceding the visit
- Legally incapable
- Chronic infectious and noninfectious lung disease except asthma (eg, pulmonary fibrosis, bronchiectasis, cystic fibrosis, tuberculosis)
- Resection of at least one lobe (or performing procedures to reduce lung volume)
- Any cancer; receiving a course of radiation or chemotherapy at the time of the visit
- Suspected lung cancer (presence of significant lung neoplasm)
- Presence of metal in the chest
- Ophthalmic surgery within the last 12 months prior to the visit
- Myocardial infarction or other form of acute or chronic coronary insufficiency or cardiac arrhythmia diagnosed at least 6 months prior to the visit
- Myocardial infarction or other form of acute or chronic coronary insufficiency or cardiac arrhythmia for which an individual regularly receives medication
- Severely elevated blood pressure (equal to or greater than a systolic 180 or diastolic of 100)
- History of cerebrovascular accident
- Thoracic or abdominal surgery within the last 6 months
- Contraindications to use salbutamol or its analogs
- CT scan or other research using ionizing radiation within the last 6 months

### Sampling

According to the Burden of Obstructive Lung Disease (BOLD) protocol, a minimal sample size of 600 is recommended to achieve an acceptable level of precision for estimating COPD prevalence [29]. Our goal is for a sample size of 900 including 500 smokers, 200 ex-smokers, and 200 never-smokers. We have used the National Health and Nutrition Examination Surveys data to assume COPD prevalence in these 3 groups [30]. The sample size of 500 allows for achieving the precision of 3.5% with the expected prevalence of obstructive impairment of 20%. The sample size of 200 and the expected prevalence of pulmonary obstruction of 10% and 2% among ex-smokers and never-smokers, respectively, provides sample estimates with 4.2% and 1.9% precision, respectively [31].

The 3 study groups are planned to have the same gender and age distribution by implementing age-gender quota to eliminate age and gender potential confounding effect to the associations between smoking status and outcomes.

It was shown that the sample characteristics depend on recruitment method used [32]. To recruit study participants, active and passive approaches are used. Participants are recruited by using personal networks of investigators and persons who are already participating in the study (snowball), placing an advertisement of the study in social media (Facebook) targeting those individuals who are potentially eligible to participate (live in Almaty, specific age group), and meeting with the owners and managerial staff of several large companies located in Almaty to explain the benefits of participation in the study. Employees of these companies who are potentially eligible to participate in the study are contacted by phone calls.

### Study Procedures

**Figure 1:**
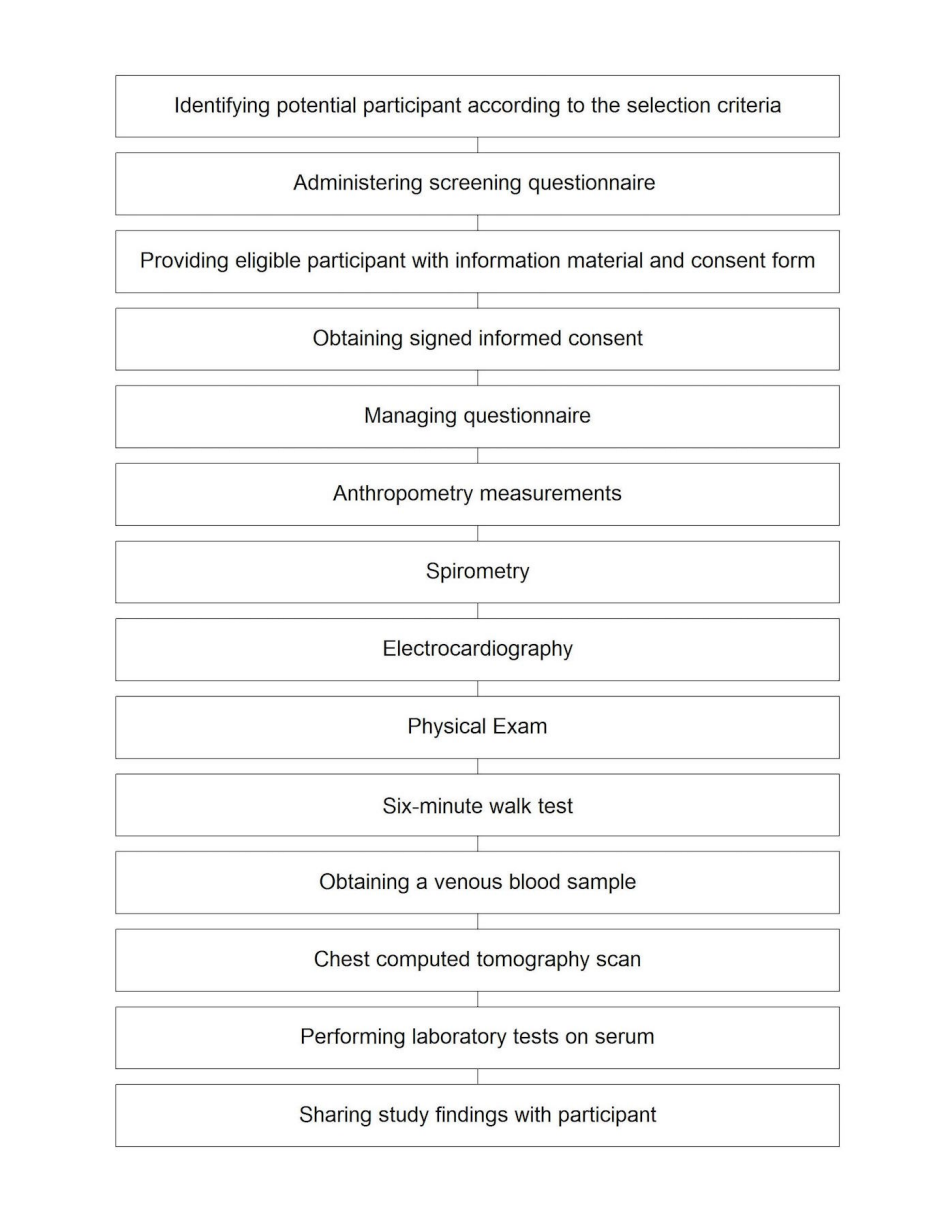
Patient recruitment, enrollment, and data collection and sharing.

The study flow is shown in  Figure 1. Participants are expected to attend 2 to 3 visits for the study, for a total of about 3 hours. Completing study procedures for each participant, including sharing the study results with the participant, is expected to take up to 2 weeks.

#### Spirometry

Spirometry is performed by trained investigators, with 3 technically satisfactory maneuvers performed by each study participant. The largest value of FEV_1_ and the respective value FVC are selected to calculate ratio of FEV_1_ to FVC. The CardioTouch 3000-S (BiTech Medical Corp), a 12-lead resting electrocardiogram (ECG) machine with a spirometer with a measuring accuracy that complies with American Thoracic Society requirements [33], is used to perform lung function tests. Calibration of the spirometer was performed every day before using it. A 3-L syringe was pumped through to check that accuracy did not exceed a tolerance of 3% [33].

#### Computed Tomography

Study subjects undergo 64-channel CT scans of the chest (Philips Brilliance CT 64). Subjects with restrictive (FEV_1_/FVC from 70% to 80%) and obstructive (FEV_1_/FVC less than 70%) lung diseases or signs and symptoms of COPD found during the physical examination and survey undergo inspiratory-expiratory CT scans obtained at the following settings: detector collimation 0.6-0.75 mm, 0.625-0.9 mm reconstructed slice thickness, 0.45-0.625 mm slice interval, 120 kV, 200 (inspiratory) and 50 (expiratory) mAs. Other subjects have only inspiratory CT scans: 0.8 mm reconstructed slice thickness, 0.4 mm slice interval, smooth reconstruction algorithm iDose 7, matrix size 512×512, range = –500 to 1500, 120 kV, 40 mAs [34].

CT scans are evaluated by 3 independent qualified observers to produce semiquantified measures that characterize the extent of emphysema, severity of bronchial dilatation, traction bronchiectasis, bronchial wall thickening, and small airway disease. We use the Bhala scoring system [35] modified by Tulek at al [36]. The kappa test will be used to evaluate interrater reliability of visual CT scan analysis. The median score by 3 observers will be recorded for each participant.

#### Electrocardiography

A standard 12-lead ECG is performed with the CardioTouch 3000-S for each study subject by employing strictly standardized procedures. Research staff members were trained to properly place electrodes. At least 4 cardiac cycles are taken from each of 12 leads. The machine runs at 50 mm/sec. The following ECG parameters are evaluated by a trained clinical researcher: waves and complexes, presence and description of ECG abnormalities including pathologic q-waves, ST elevation, ST depression, T-wave inversion, hypertrophy, QRS axis deviation, block, and arrhythmia. ECGs were visually inspected for technical errors and were interpreted by a qualified cardiologist. The prevalence of specific ECG abnormalities as well as grouped abnormalities will be reported for each study group. Associations between COPD and ECG abnormalities, crude and adjusted by sex, age, and smoking status, will be measured and presented.

#### Physical Exam

Clinical investigators were trained to conduct the pulmonary (percussion and inspection) exam and technique for listening to second heart sounds. Two Stanford Medicine 25 modules were used as study materials in hands-on sessions [37,38]. The prevalence of individual pathological findings will be presented for each study group. Associations between pathological findings from 2 exams and COPD will be evaluated.

#### Anthropometry

Anthropometry measures include height, weight, waist circumference, heart rate, blood pressure, and pulse oximetry.

#### Six-Minute Walk Test

All study subjects will take a 6-minute walk test to evaluate functional exercise capacity. Investigators assess whether contraindication exists or not. Absolute contraindications are unstable angina and myocardial infarction during the previous month. Relative contraindications are blood pressure more than 180/100 mm Hg and a resting heart rate of more than 120 beats per minute [39]. Subjects with any of the contraindications are referred to the clinical coordinator for a decision about the conduct of the test. Posttest dyspnea is measured using the Borg scale [40].

#### Laboratory Data

Serum from each participant is tested for blood cholesterol level, high-density lipoprotein (HDL), low-density lipoprotein (LDL)), triglycerides, C-reactive protein, fibrinogen, glucose, hepatitis B and C IgM and IgG antigens and antibodies, alanine aminotransferase (ALT) and aspartate aminotransferase (AST) liver enzymes, and α_1_-antitrypsin.

#### Questionnaire

The questionnaire was designed to collect data on possible COPD risk factors including history of smoking; current smoking; level of smoking exposure (in pack-year); passive smoking; and occupational and environmental hazards including dusts, chemicals, and indoor fuel pollution. The questionnaire contains the following covariates: age, gender, ethnicity, education, occupation, and self-reported morbidity. CAT is used to collect Information about COPD symptoms. The test was designed and validated for use in routine clinical practice to evaluate the health status of patients with COPD [41]. Computer-assisted personal interviewing (CAPI) with tablets has been implemented to collect, store, and transmit data related to a personal survey interview.

The questionnaire was piloted by interviewing 7 testers—3 smokers, 2 ex-smokers, and 2 never-smokers. After completing pilot interviews, the testers were asked to answer specific questions and then provide comments and suggestions on the whole questionnaire. The questions that were not clear enough were identified and have been improved.

### Statistical Analysis

Data from all CAPI devices will be exported to one database, and R version 3.3.1 (The R Foundation) will be used for data analysis. Descriptive analyses will be performed using mean, median, interquartile interval, and standard deviation for quantitative variables and frequency tables for categorical variables. Depending on the nature of outcome and exposure variables, type of data distribution, and the sample size, bivariate comparisons will be made by the following tests: chi-square test, Fisher’s exact test, analysis of variance, *t* test, and the Mann-Whitney test.

A multivariable analysis (generalized linear models) will be conducted to control for confounding. Presence of effect modification/interaction terms will be explored. An alpha <.05 will be considered significant. To construct the optimal model, backward elimination will be used. The full models will contain all independent effects and some important interactions. Mediators (variables that lie on the causal pathway between exposure and outcome) will not be included in the model. To avoid multicollinearity, we will examine the tolerance for each independent variable. If the tolerance value is less than 0.1, we will omit a variable from the analysis. The optimal model will be defined based on the Akaike Information Criterion. Model diagnostic plots will be generated to test model assumptions (eg, normality of deviance residuals).

### Ethics Approval

The National Central Ethics Committee under the Ministry of Health and Social Development in the Republic of Kazakhstan approved this study on August 19, 2016. The study has been registered at ClinicalTrials.gov [NCT02926534].

## Results

We have completed the participant recruitment and study procedures. Currently, we are working on data processing and data analysis. The authors anticipate the preliminary results should be available by September 2017. Study results will be published in peer-reviewed scientific journals.

## Discussion

To the best of our knowledge, this is the first study in Kazakhstan that assesses COPD prevalence in the general population aged 40 to 59 years, specifically smokers, ex-smokers, and never-smokers. The study also aims to investigate COPD comorbidities and their interaction with COPD. Improving COPD preventive measures, COPD screening, identification, and registration requires obtaining this epidemiological information. Study strengths include its relatively large sample size and collection of comprehensive medical, behavioral, and other health-related data from each study participant.

However, there remain some limitations. First, the study is observational. Thus, the possibility of unidentified or unmeasured confounders exists. We are going to conduct the sensitivity analysis to assess a covariate that could eliminate the effect of smoking on COPD. Second, the study is cross-sectional, and the time when COPD occurred cannot be identified. The values of potential confounders measured in the study could differ from ones when symptoms of COPD first appeared and could be diagnosed. As a result, causal inferences cannot be made. Third, the study includes many components to be measured, which makes it impossible to employ probability sampling to select participants. The main risk of nonprobability sampling is that the distribution of important covariates in the sample will differ significantly from their distribution in the study population. We employ quota sampling to set quotas for gender and age, 2 important covariates for COPD, to balance them in the sample. In addition, we measure all known important covariates or confounders to make the desired adjustments in our data analysis. Fourth, the study is conducted in one city, Almaty, which reduces the generalizability of the study results to the country population. Finally, we expect some measurement bias that arises from errors in the data collection. For example, participants could avoid socially undesirable answers. To reduce the latter bias, all data collection procedures were tested and the research staff members were trained.

## Abbreviations

BOLD: Burden of Obstructive Lung Disease
CAT: COPD Assessment Test
CAPI: computer-assisted personal interviewing
COPD: chronic obstructive pulmonary disease
CT: computed tomography
ECG: electrocardiogram
FEV1: forced expiratory volume in 1 second
FVC: forced vital capacity
